# Effects of the ghrelin receptor agonist anamorelin on lean body mass in cancer patients with cachexia; results from a Phase II randomized, double blind, multicenter study

**DOI:** 10.1186/2049-3002-2-S1-P19

**Published:** 2014-05-28

**Authors:** Jose M Garcia, Ying Yan, Elizabeth Manning-Duus, John Friend

**Affiliations:** 1 Department of Medicine, Molecular & Cellular Biology, and Huffington Center on Aging, Baylor College of Medicine, Houston, Texas, USA; 2 Center for Translational Research in Inflammatory Diseases, Michael E De Bakey VA Medical Center, Houston, Texas, USA; 3 Helsinn Therapeutics, Inc., Bridgewater, New Jersey, USA

## Background

Cancer anorexia-cachexia is a frequent, debilitating and life-threatening condition in which altered metabolism and reduced food intake contribute to weight loss (mainly due to lean body mass [LBM] loss), which cannot be reverted by conventional nutritional support. Safe/effective treatments for cancer cachexia remain an unmet need. The hunger hormone ghrelin has been shown to activate key pathways in the regulation of body composition. Anamorelin (ANAM) is a novel, selective, oral ghrelin receptor agonist with appetite-enhancing and anabolic activity. Anamorelin is currently being investigated in phase III studies for the treatment of anorexia-cachexia in advanced non-small cell lung cancer (also known as the ROMANA program).

## Material and Methods

Eighty-two patients with advanced cancer, ECOG performance score ≤ 2 and weight loss 5% within prior 6 months were treated with placebo (n=36) or 50mg anamorelin (n=38) once daily for 12 weeks. Body composition by DXA, handgrip strength (HGS) and Quality of Life (QoL; ASAS scale) were assessed at baseline, 4, 8 and 12 weeks. Solid tumors represented prevalent malignancy (>93%) with no difference in cancer types between treatment groups.

## Results

Treatment with anamorelin for 12 weeks significantly increased total body mass (TBM) and LBM. Increases in LBM and TBM in anamorelin-treated patients were strongly correlated (r^2^=0.7249, p< 0.0001). Anamorelin treatment improvements in HGS and QoL were previously presented [1]. Anamorelin was well tolerated, and types and prevalence of AEs were similar between treatment arms.

## Conclusion

Decreased body weight and LBM are poor prognostic factors in cancer cachexia patients. This study demonstrates that 50mg anamorelin treatment for 12 weeks significantly increased LBM, which largely contributed to the increases in total body mass. Together with its appetite-enhancing activity, these results support the further development of anamorelin in treating cancer anorexia-cachexia.
